# Molecular Mechanisms in the Vascular and Nervous Systems following Traumatic Spinal Cord Injury

**DOI:** 10.3390/life13010009

**Published:** 2022-12-20

**Authors:** Shuo Li, Hoai Thi Phuong Dinh, Yukihiro Matsuyama, Kohji Sato, Satoru Yamagishi

**Affiliations:** 1 Department of Organ and Tissue Anatomy, Hamamatsu University School of Medicine, Hamamatsu 431-3192, Japan; 2 Department of Orthopedic Surgery, Hamamatsu University School of Medicine, Hamamatsu 431-3192, Japan; 3 Optical Neuroanatomy, Preeminent Medical Photonics Education & Research Center, Hamamatsu University School of Medicine, Hamamatsu 431-3192, Japan

**Keywords:** spinal cord injury, blood-spinal cord barrier, glial scar

## Abstract

Traumatic spinal cord injury (SCI) induces various complex pathological processes that cause physical impairment and psychological devastation. The two phases of SCI are primary mechanical damage (the immediate result of trauma) and secondary injury (which occurs over a period of minutes to weeks). After the mechanical impact, vascular disruption, inflammation, demyelination, neuronal cell death, and glial scar formation occur during the acute phase. This sequence of events impedes nerve regeneration. In the nervous system, various extracellular secretory factors such as neurotrophic factors, growth factors, and cytokines are involved in these events. In the vascular system, the blood-spinal cord barrier (BSCB) is damaged, allowing immune cells to infiltrate the parenchyma. Later, endogenous angiogenesis is promoted during the subacute phase. In this review, we describe the roles of secretory factors in the nervous and vascular systems following traumatic SCI, and discuss the outcomes of their therapeutic application in traumatic SCI.

## 1. Introduction

Traumatic spinal cord injury (SCI) induces various complex pathological processes that cause physical impairment, psychological devastation, and social burdens [1,2].

In terms of its pathophysiology, traumatic spinal cord injuries are usually divided into primary and secondary injuries. Secondary injuries include acute-(approximately 48 h after injury), subacute-(about 14 days after injury), and chronic phases (approximately 6 months or more after injury) [3]. After injury, the microenvironment develops differently in each phase because of the presence of many blood vessels and neurons in the spinal cord. During the acute phase, destruction of spinal cord microvasculature causes cell degeneration and necrosis, and a large number of cytokines are released from inflammatory cells and infiltrate the injury site [4,5,6]. These events involve an ionic imbalance, excitotoxicity, calcium influx, free-radical production, lipid peroxidation, inflammation, edema, and necrotic cell death [7,8,9].

The subacute phase is defined as lasting for up to 2 weeks, during which the cellular microenvironment of the injured spinal cord is dramatically altered. Astrocytes, microglia, and NG2 cells are stimulated by cytokines and chemokines, and proliferate and migrate toward the lesion site [10,11,12,13]. Meanwhile, hematogenous macrophages and fibroblasts begin accumulating in the lesion core [14,15]. The astroglial and fibrotic scars formed by the two groups of cells tend to be stable for 14 days after the injury. During the chronic (>14 days) phase, alterations in the microenvironment of the spinal cord slow down. When hemorrhaging stops, the organism enters a state of self-repair, attempting to regenerate myelin and axons as well as vascular reorganization [16,17,18]. At the lesion site, an escalating injury cascade, including vascular disruption, inflammation, demyelination, and apoptosis, leads to the formation of glial scars and cavities [19,20]. Ruptured blood vessels are essential for nerve regeneration and functional recovery. Furthermore, it is important that the tissue is adequately vascularized so that oxygen, nutrients, and metabolic wastes are delivered and removed.

Angiogenesis is one of the early forms of healing but does not eventually lead to the spontaneous restoration of injury-induced anatomical and functional damage. Blood vessels that have ruptured or are dysfunctional (i.e., leaking) have major roles in the progressive nature of tissue loss and the inability of nerve tissue to heal in the spinal cord [21,22,23]. Blood vessels that have ruptured at the site of the injury cause hemorrhages, which accelerate the loss of tissue both immediately and over time. Limiting the early deterioration of blood vessels may aid healing of the spinal cord. Although endogenous angiogenesis occurs at the site of the injury, it does not provide sufficient vascularization, which inhibits the body’s natural healing process as well as restorative therapies.

**Figure 1:**
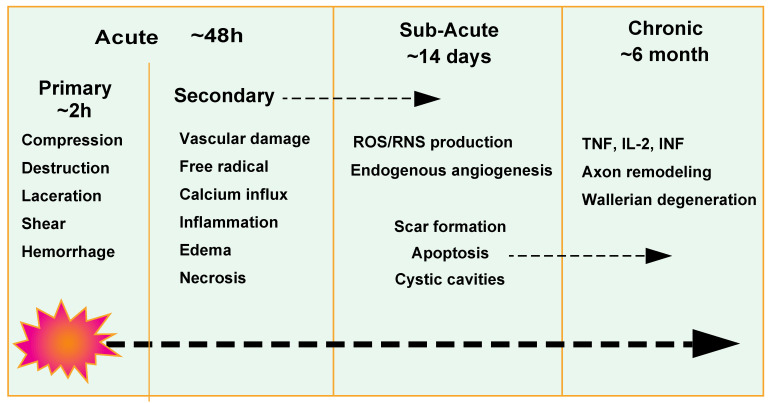
Pathophysiology of traumatic-spinal cord injury.

The BSCB, a structure that is anatomically similar to the blood–brain barrier, becomes increasingly permeable and leaky, not only in damaged areas but also in the surrounding tissues [24]. This deterioration exacerbates pre-existing tissue damage by inducing ischemia and inflammation [25]. Angiogenesis is inadequate even when the lesion generates new blood vessels. Therefore, it is crucial to understand the vascular reactions that occur in the microenvironment of the lesion after SCI. To treat SCIs, interventions that regulate vascular responses are essential to promote the formation of an adequate number of functional vessels [26]. In this review, we will provide an overview of the molecular changes that occur in the nervous and vascular systems following SCI (Figure 1).

## 2. Molecular Mechanisms in the Nervous System

The microenvironment in the nervous system is altered at the molecular level after spinal cord injury, and various neural repair-related molecules are highly expressed, while some injury-related molecules that affect the body’s recovery are also abundant. In this section, we described the responses in the nervous system after spinal cord injury, focusing on neurotrophic factors, growth factors, cytokines, and regulatory-related ions.

### 2.1. Neurotrophic Factors

**Figure 2:**
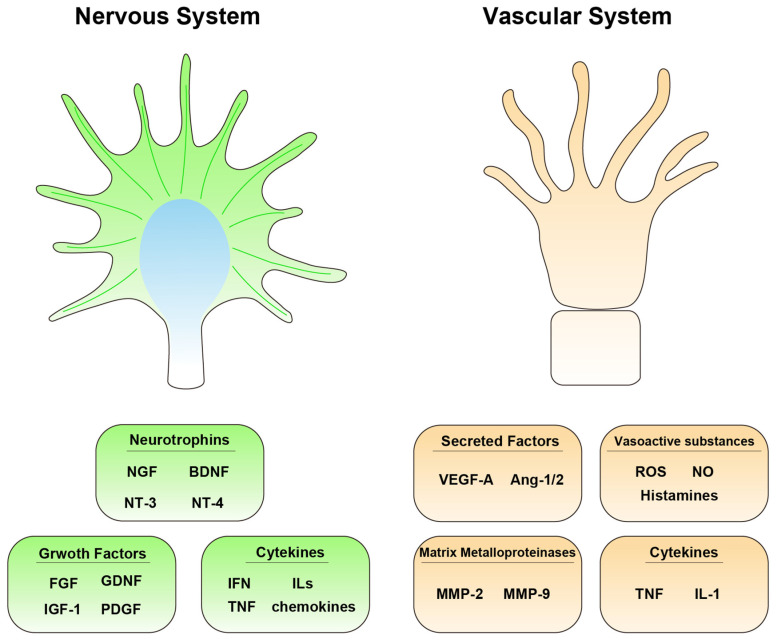
Extracellular molecules involved in the responses after spinal cord injury in the nervous and vascular systems.

Neurotrophic factors, including nerve growth factor (NGF), brain-derived neurotrophic factor (BDNF), neurotrophin-3 (NT-3), and NT4, are involved in the development and maintenance of the nervous system. Neurotrophic factors aid in rebalancing of the microenvironment after SCI [27,28] (Figure 2; Table 1).

NGF was the first neurotrophic factor to be identified [29]. Mature NGF in the central nervous system (CNS) is neuroprotective, and binds to TrkA and p75 NTR receptors to activate downstream signaling cascades that promote neuronal differentiation and survival. Conditional expression of NGF in the adult rat spinal cord increases axonal sprouting of sensory afferent nerves and improves behavioral outcomes [30].

BDNF exerts neuroprotective and growth-promoting effects on a variety of neuronal populations after injury [28]. Its neuroprotective effects, in particular, are attributed to the downstream effects of TrkB receptor signaling. BDNF also promotes the regeneration and formation of damaged axons in the spinal cord and the regeneration of myelin in damaged axons [31,32]. TrkB receptors are expressed in many neurons in the spinal cord [28]. The high expression of TrkB in spinal cord neurons sometimes produces detrimental effects, since pain and spasticity are sometimes positively associated with the administration of BDNF. Interventions can be accomplished by blocking BDNF-TrkB signaling [33,34]. The precursor of BDNF (proBDNF) is also expressed and has an impact following SCI. ProBDNF induces neuronal death via the p75^NTR^ [35] in vitro, and similarly, antibody treatment with proBDNF promotes neuronal survival and improves functional recovery after SCI [36].

NT-3, another member of the family of neurotrophic factors, is highly expressed in motor neurons in the developing spinal cord [37]. However, NT-3 expression is very low in the mature spinal cord. After SCI, NT-3 expression decreases rapidly during the acute initial phase but thereafter returns to normal levels [38]. NT-3 binds with low affinity to TrkA, TrkB, and p75 NTR receptors but has the highest affinity for TrkC receptors that are expressed by neurons in the corticospinal tract (CST) [39]. NT-4 is a little-studied neurotrophic factor. NT-4 expression is increased after SCI. A previous study showed that NT-4 may automatically regulate further expression of NT-4 and TrkB receptors in phrenic motor neurons after SCI, and that NT-4 promotes the functional recovery of the diaphragm after cervical SCI [40].

Those neurotrophic factors are mostly acting on neurons rather than other cell types, which often improve functional recovery after injury by promoting the axonal growth and branching.

### 2.2. Growth Factors

Growth factors include fibroblast growth factors (FGF), glial cell-derived neurotrophic factor (GDNF), insulin-like growth factor (IGF-1), and platelet-derived growth factor (PDGF). The expression levels of all of these factors change after SCI and affect the spinal cord microenvironment and the recovery of motor function (Figure 2; Table 1).

Among the FGF family of proteins, basic FGF (bFGF) is the best characterized in SCI. Endothelial cells, smooth muscle cells, and macrophages secrete bFGF, and bFGF is thought to promote lesion formation. Studies on the effect of FGF in SCI reveal improvements in the spinal cord microenvironment after FGF treatment, and several studies have reported improvements in motor function [41,42,43,44]. Furthermore, FGF reduces glial proliferation and increases the number of radial glial and neural progenitor cells, indicating a close association between FGF and reactive glial cells [45].

GDNF is expressed in different regions of the CNS. Specific cellular sources of GDNF are type I astrocytes, the substantial nigra-striatal system, and neurons of the basal forebrain [46]. GDNF is the most potent cholinergic motor neurotrophic factor and supports the survival of motor neurons. In models of SCI, GDNF treatment prevents spinal cord motor neuron death and atrophy, and improves locomotor function [47,48,49,50,51]. Therefore, GDNF has the potential to be used in neural transplantation or in neural regeneration.

IGF-1 specifically enhances the axonal growth of corticospinal motor neurons through a PI3-dependent pathway [52]. Gene transfer of IGF-1 is neuroprotective and improves locomotor function after SCI [53].

PDGF is a proangiogenic factor isolated from platelets. Its receptor is a member of the tyrosine protein kinase family, which promotes cell chemotaxis, division, and proliferation, and plays active and important roles in the physiological processes of growth, development, and the repair of cellular damage. The binding of PDGF and NT-3 to transplanted fibronectin scaffolds increases neuronal survival and differentiation in a subacute model of SCI [54].

The other two classes of growth factors, ciliary neurotrophic factor (CNTF) and leukemia inhibitory factor, were first studied when examining the regeneration of axons from optic nerve system injuries; however, their roles in SCIs are rarely mentioned [55,56]. CNTF is found in reactive astrocytes that are expressed after SCI and, together with bFGF, produces effects on glial scar formation [41]. We recently reported that the axon guidance molecule FLRT2 is upregulated after SCI, and that glial scar formation is inhibited in FLRT2 conditional knockout mice [57]. Since FLRT2 is a repulsive guidance molecule, FLRT2 inhibition will have dual benefits in terms of attenuating repulsion and preventing scar formation [58].

Those molecules are tightly associated with glial scarring after SCI. Because of their roles in promoting glial cell growth and differentiation, they often play an active role in the microenvironment of the spinal cord.

### 2.3. Cytokines

Cytokines involved in SCI include interferon (IFN), interleukins (ILs), tumor necrosis factor (TNF), and chemokines. Cytokines are key regulatory proteins in the immunoinflammatory cascade and cellular repair system [59] (Figure 2; Table 1).

IFNs are produced by lymphocytes in the human immune system in response to viral stimulation and have some antiviral effects. There are many subtypes of IFNs in humans. The first IFNs to be discovered were α, β, and γ [60]. IFNs α and β are classified as type I, and IFN γ is classified as type II. The recently described type III IFNs include IFN-λ1, IFN-λ2, IFN-λ3, and IFN-λ4 [61,62]. Studies of IFNs in SCI have focused on IFN-β and IFN-γ, both of which improve lower extremity locomotor function after the injury [63,64]. Treatment with IFN-β-injected neural stem cells reduces astrocyte proliferation and increases axonal retention, ultimately improving motor function [65]. IFN-γ treatment induces the strong expression of CD11b+ macrophages/microglia in the spinal cord, which are concentrated at areas bordering the injury site. IFN-γ treatment increases astrocyte activation. In the injured spinal cord, the levels of chondroitin sulfate proteoglycan, an inhibitor of axonal regeneration produced by astrocytes, are significantly reduced, whereas the levels of GDNF and IGF-I mRNA are increased [64,66].

ILs are cytokines produced and secreted by many immune and nonimmune cells. They are originally thought to be produced by leukocytes and directly contribute to the growth and development of immune cells [59]. The IL family is large and diverse, and exhibits a wealth of functions in SCI. TNF, one of the most widely distributed and earliest appearing factors during the inflammatory response, acts synergistically with members of the IL family to trigger the inflammatory response. TNF and ILs often appear together after SCI. At the start of the acute phase in SCI, microglia/macrophages, astrocytes, and neurons synthesize the proinflammatory cytokines IL-1β, TNFɑ, and IL-6. These cytokines are considered to be the major players in the early injury timeline and remain present beyond the subacute phase [5]. IL-6 inhibition promotes SCI repair by inducing microglia-based inflammation, while TNFα signaling is neuroprotective in vitro and promotes functional recovery after SCI [67,68]. IL-4 and IL-13 are anti-inflammatory-related cytokines that induce alternative macrophage activation [69,70]. IL-10, another anti-inflammatory cytokine, downregulates proinflammatory cytokines levels [71]. Continuous treatment with IL-10 reduces inflammation and improves motor function after SCI [72].

Chemokines are a class of small cytokines or signaling proteins secreted by leukocytes and various other cells involved in the inflammatory process. Based on the amino acid sequence inserted between the first two CC amino acids in the sequence, there are four categories of chemokines: C, CC, CXC, and CX3C. CC chemokines are associated with the activation and migration of monocytes and lymphocytes, whereas CXC chemokines are often linked to the activation and migration of neutrophils and macrophages [73]. After SCI, the CC chemokine subtype CCL2 is protective by recruiting macrophages to the site of the injury and promoting the conversion of macrophages into an anti-inflammatory, neuroprotective M2 phenotype. Treatment with CCL2 prevents motor neuron degeneration and improves motor function [74]. However, some catalytic factors produce the opposite effect, with CCL20-neutralizing antibodies reducing edema and inflammation after SCI and improving motor function [75]. It is evident that chemokines are a double-edged sword in SCI, and that excessive immune cell activation can lead to inflammation-driven injury.

The presence of cytokines was always associated with the inflammatory reaction, different cytokines play different roles in SCI recovery by activating the pro- and anti-inflammatory effects of the bodies.

### 2.4. Regulatory-Related Ions

Changes in ion channels after SCI are an important cause of regulatory-related ions impacting SCI. Ion channel blockers have been used to study the imbalance in ion homeostasis after injury. The most common ions after SCI are Na^+^, K^+^, Ca^2+^, and Fe^2+^, all of which are upregulated in the spinal cord in response to SCI [76,77]. Studies using Na^+^ channel blockers (e.g., tetrodotoxin, riluzole, and phenytoin) demonstrate that inhibition of Na^+^ channels is neuroprotective when treating SCI [78,79,80,81]. Treatment with the K^+^ channel blocker 4-aminopyridine (4-AP) decreases astrocyte proliferation and myelin regeneration and improves neuromuscular function [82,83]. Ca^2+^ plays a crucial role in CNS injury. Ca^2+^ concentrations increase rapidly after SCI, reach a peak during the preacute phase, and remain high until the end of the subacute phase. High concentrations of intracellular Ca^2+^ cause apoptosis or necrosis through increased activation of cellular enzymes, mitochondrial damage, acidosis, and free-radical production [77,84]. Ca^2+^ permeable activation of acid-sensing ion channel 1a (ASIC1a) channels is increased after SCI, and inhibition of ASIC1a promotes functional recovery in animals with SCI [85]. Ferroptosis is a form of cell death characterized by the demand for iron and the accumulation of reactive oxygen species (ROS) [86]. Inhibition of ferroptosis induced by iron overload via iron death inhibitors, such as desferrioxamine (DFO), N-acetylcysteine (NAC), and ferrostatin-1 (Fer-1), attenuates neuronal death and promotes the integrity of motor pathways and motor recovery after SCI [87,88].

The injury of the spinal cord is accompanied by the imbalance of ions, and the general upregulation of various ions may causes apoptosis. The inhibition of these ions plays a therapeutic role in SCI.

## 3. Molecular Mechanisms in the Vascular System

The vascular system is severely disrupted after spinal cord injury and its microenvironment is more complex than that of the nervous system. The constant influx of vascular and blood-related molecules makes the vascular system have different and complex molecular mechanisms in each injury phase. In this section, we investigate the mechanisms of vascular-related molecules and their effects on spinal cord repair by examining the manifestations of the vascular system at different stages after spinal cord injury. These include vascular response after spinal cord injury, blood vessel loss, blood spinal cord barrier breakdown, endogenous angiogenesis, and spinal cord recovery (Figure 1).

### 3.1. Vascular Responses after Spinal Cord Injury

Each of the interconnected events that make up the SCI pathophysiology acts as a trigger for the pathophysiology of the others. Several events may occur simultaneously, producing complex features of the disease, thereby making it challenging to treat. SCI may be seen as a cascade of many interconnected SCI pathophysiological events, each of which acts as a catalyst for the events that follow [3]. The disruption of blood supply causes hypotension/hypoperfusion, hypovolemia, neurogenic shock, and bradycardia after the injury. These symptoms develop because of significant bleeding and neurogenic shock, which cause spinal cord ischemia. Leukocytes and red blood cells are more likely to extravasate when tiny blood arteries and capillaries burst. These immune cell extravasations at the injury site place pressure on the injured spinal tissues and further obstruct blood flow, causing a vasospasm [89]. This condition may last for up to 24 h, and vascular ischemia, hypovolemia, and hyperperfusion damage occur [89,90]. Thus the disruption of blood flow to the lesion, simultaneously with the activity of immune cells at the ruptured vessel causes a chain of pathological activities of the spinal cord injury.

### 3.2. Blood Vessel Loss

The degree of damage from a SCI is directly connected to the rupture of the local microvasculature structure and the separation of blood vessels from astrocytes [91]. The blood vessel wall then splits from the BM into an exterior parenchymal part and an interior endothelial part [92]. Necrosis causes substantial loss of endothelial cells (EC) within the first 24 h after the injury. Then, in the days that follow, ECs undergo apoptosis, which is mostly caused by ischemia. At the epicenter of the lesion, insufficient blood supply also causes neuronal apoptosis and cell death [93,94].

The density of blood vessels surrounding the lesion continues to decrease. During the subacute phase of SCI, structurally altered BMs further accelerate the spread of inflammation. Red blood cell lysis, extracellular glutamine levels, and iron toxicity all increase because of the bleeding, which hastens thrombin generation and aggravates axonal injury [94]. Therefore, blood vessel loss consists of the event of rupture of microvascular structures, and according to the course of the period from acute to, necrosis causes loss of endothelial cells leading to apoptosis process, which reduces the density of blood vessels around the damage significantly, in the area of gray matter and white matter, with bleeding generally begins in the gray matter but eventually spreads radially into the adjacent white matter [94,95].

### 3.3. Blood Spinal Cord Barrier Breakdown

Spinal cord homeostasis is supported by the BSCB, a tight barrier between the blood and spinal cord tissues [96,97]. The neurovascular unit and membrane structure are disrupted by the initial mechanically damaging force in concert with compression or vascular dilation-induced shear stress [98,99]. Vascular permeability is increased via injury-induced proinflammatory cytokines (e.g., TNF and IL-1), vasoactive substances (including reactive oxygen species, nitric oxide, and histamines), and matrix metalloproteinases (MMPs) [100,101]. BSCB disruption occurs rapidly within the first several hours. Infiltrating immune cells, such as lymphocytes, neutrophils, and monocytes, trigger an inflammatory reaction at the site of the lesion. Secondary damage following SCI is exacerbated by the entry of calcium, excitatory amino acids, free radicals, and inflammatory mediators into the injury site [26].

Collapse of the BSCB through hemodynamic, structural, or chemical changes caused by SCI frequently results in inflammation and the influx of hazardous chemicals such as calcium, excitatory amino acids, and free radicals. Interestingly, the rupture of pre-existing blood arteries is often required to facilitate an angiogenic response, which results in increased permeability. Molecules including VEGF-A, Ang2, and matrix metalloproteinases (MMP)-2 and -9 promote BSCB collapse, whereas Ang1 supports BSCB stability [102]. The caveolae, Cav-1, and tight junction proteins, which constitute the majority of the BSCB, are all implicated in the breakdown of this barrier.

With the rupture of BSCB after spinal cord injury, affects the mechanical structures between the blood vessels and nervous tissue, thereby causing angiogenesis especially VEGF, increasing permeability and stimulating the inflammatory response (Figure 2; Table 1).

### 3.4. Endogenous Angiogenesis

With stimulation of growth factors in the chain of chemical reactions that occur around the damage, the formation of blood vessels occurs, through three primary processes, vasculogenesis, splitting angiogenesis, and sprouting angiogenesis, are involved in the generation of blood vessels [103]. Angiogenesis is the primary mechanism of blood vessel formation in the lesion microenvironment after SCI. Proangiogenic growth factors and hypoxia cause ECs to sprout, proliferate, and finally remodel. Within 2 weeks, endogenous angiogenesis briefly increases the density of blood vessels. However, endogenous angiogenesis is insufficient for maintaining local metabolism, which hastens hypoxic ischemia and cell death at lesion sites. Angiogenesis and endothelial regeneration include several chemicals and signaling pathways [104,105]. EC proliferation is aided by transcription factors such as FoxM1 [106], HIF-1α [107], Sox17 [108], Atf3 [109], and Foxo1 [110]. Mef2 elements regulate sprouting angiogenesis [111]. These transcription factors interact with several signaling pathways to modify angiogenesis. VEGF signaling in angiogenic processes occurs in both healthy and pathological states [112]. PI3K-Akt signaling activation stimulates VEGF expression, HIF-1α synthesis, and angiogenesis [99]. It is possible to activate Foxo1 and induce EC elongation by blocking the PI3K-Akt and mTOR signaling pathways [113].

With the formation and regeneration of vessels through signaling pathways led to events and interactions between blood vessels and nerves to ensure the integrity and recovery of neural vessels after spinal cord injury. Furthermore, newly formed blood vessels with a deficient BSCB are prone to leaking [21]. New arteries fail to become functional by forming an association with other cells (neurons, astrocytes, or pericytes) [26,114]. The role of the VEGF and Ang protein families in maintaining BSCB homeostasis is gaining popularity. These chemicals have been termed “angioneurins” because it is now clear that they have an effect on neurons as well as on several angioneurins that may influence the integrity of the BSCB [115].

### 3.5. Spinal Cord Recovery

Delivery of proangiogenic agents, gene regulation, and other vascular interventions are carried out as part of the development of vascular responses and augmentation of angiogenesis processes in SCI [116]. Thus, a series of interactions between blood vessels and nerves in the molecular signaling pathway have promoted and supported many of the treatments that support this association (Table 1).

(i) Administration of proangiogenic factors: VEGF has a considerable impact on EC migration and proliferation and on blood vessel development [43]. In various trials, alone or in combination, treatment with VEGF and its isoform (VEGF-A165, 121, 189) significantly improved neuroprotection and post-traumatic recovery. Furthermore, the administration of modified zinc protein transcription factor (ZFP) activates all VEGF-A isoforms, whereas VEGF combined with PDGF, bFGF, and Ang1 increases blood vessel density, decreases BSCB permeability, and increases blood supply [116]. Several hormones, enzymes, or chemicals, including melatonin and estrogen, influence angiogenesis in the treatment of SCI. Chondroitinase ABC (ChABC) promotes axonal remodeling and regeneration by inducing revascularization. ChABC causes the degradation of extracellular chondroitin sulfate proteoglycans (CSPG), promoting angiogenesis and protecting BM vessels. Furthermore, the presence of MMPs, flufenamic acid (FFA) or MMP-8 inhibitors, and granulocyte colony-stimulating factor (G-CSF) increases local revascularization and prevents BSCB disruption [117].
(ii) Gene modulation: Many studies have found evidence of neuroprotection and functional recovery via gene manipulation. Kumar et al. investigated transient potential channel protein (TRPV4) function following SCI and discovered that TRPV4 activation has negative effects on endothelial cell damage, the progression of inflammation, and rehabilitation and functional recovery [118]. The decrease in ubiquitously transcribed tetratricopeptide repeat on chromosome X (UTX) levels, a histone H3K27 demethylase that is substantially increased after SCI, promotes EC migration and tubule/tube formation/genesis via the miR-24 pathway, and epigenetically stimulates vascular remodeling and functional retrieval [119].
(iii) Cell-based therapy approaches: Stem cell transplantation has emerged as a convincing therapeutic strategy in both degenerative and traumatic illnesses because of the inherent differentiation variety and favorable treatment possibilities provided by stem cells. Mesenchymal stem cells derived from the bone marrow, umbilical cord, adipose tissue, and amnion encourage BSCB repair and improve revascularization at the location of the lesion [116].

Furthermore, therapies involving physical stimulation, such as water treadmill training in mouse models, increases EC proliferation and BSCB maintenance at the lesion site and has an angiogenic effect [120].

## 4. Conclusions

The regulation of the nervous system after SCI involves complex interactions among multiple molecules. Understanding the role of each of these molecules is important for guiding the treatment of SCI. Revascularization is generally necessary for recovery after SCI. Extensive knowledge of all of the intrinsic mechanisms that occur in primary SCI is a prerequisite for successful therapy and rehabilitation. The rupturing of blood vessels after SCI and the molecular interaction between blood vessels and nerves are particularly important. However, numerous aspects of this process remain unidentified, and more in-depth research is required to develop appropriate treatment strategies. Clinical studies using secreted factors, as discussed in this article, to aid regeneration of the nervous system and promotion of angiogenesis should be pursued further to treat SCI.

## Figures and Tables

**Table 1:** Molecules related to the vascular and nervous systems.

**Molecules in the Nervous System**	**Molecules in the Vascular System**
Neurotrophic factors NGF (+) BDNF (+), proBDNF (−) NT-3 (+) NT-4 (+) Growth factors FGF (+) GDNF (+) IGF-1 (+) PDGF (+) Cytokines IFN (+) ILs: IL-6 (−), IL-10 (+) TNF (+) Chemokines: CCL2 (+), CCL20 (−) Ions: Na^+^ (−), K^+^ (−), Ca^2+^ (−), Fe^2+^ (−)	VEGF : VEGF-A165, 121, 189 (+) Hormones Melatonin (+) Estrogen (+) Enzymes : ChABC (+) Chemicals MMPs (+) FFA (+) G-CSF (+) TRPV4 (−) UTX (−)

Positive (+) and negative (−) correlations with functional recovery after SCI.

## Data Availability

Not applicable.
